# 

**Published:** 1922-09

**Authors:** 


					CONTENTS.
Page
The Bristol Medical Officer of Health
69.
Oral Sepsis as a Source of Systemic Infections.
By W. R. Ackland,
M.R.C.S., L.D.S., M.D.S.
76
The Differential Diagnosis of Malignant Disease of the Caecum
from Chronic and Subacute Appendicitis.
By Charles A.
Morton, F.R.C.S.
82
Reviews of Books
9r
Editorial Notes
1 ?3
The Library of the Bristol Medico-Chirurgical Society
Obituary :
Edward Hugh Edwards Stack, M.B., B.Ch. Cantab.,
F.R.C.S. Eng.
Local Medical Notes     n 5

				

